# SLP2/PHB Aggregates in ALS Mouse Models and Patients: Implications Beyond *CHCHD10*-Associated Motor Neuron Disease

**DOI:** 10.3390/ijms262210852

**Published:** 2025-11-08

**Authors:** Emmanuelle C. Genin, Françoise Lespinasse, Alessandra Mauri-Crouzet, Luc Dupuis, Véronique Paquis-Flucklinger

**Affiliations:** 1 Team “Mitochondria, Diseases and Aging”, Institute for Research on Cancer and Aging, Nice (IRCAN), Inserm U1081, CNRS UMR7284, Université Côte d’Azur (UniCA), 06107 Nice, France; francoise.lespinasse@univ-cotedazur.fr (F.L.); alessandra.mauri-crouzet@univ-cotedazur.fr (A.M.-C.); paquis@unice.fr (V.P.-F.); 2 Centre de Recherche en Biomédecine, Strasbourg Translational Neuroscience and Psychiatry, UMR-S1329, Inserm, Université de Strasbourg, 67000 Strasbourg, France; ldupuis@unistra.fr; 3 Department of Medical Genetics & Reference Centre for Mitochondrial Disease, Nice Universitary Hospital, 06202 Nice, France

**Keywords:** Amyotrophic Lateral Sclerosis, SLP2/PHB aggregates, motor neuron disease, *CHCHD10*

## Abstract

Amyotrophic Lateral Sclerosis (ALS) is a fatal neurodegenerative disorder characterized by motor neuron (MN) degeneration, frequently overlapping with frontotemporal dementia (FTD). Protein aggregation is a hallmark of these disorders, yet the role of aggregates in ALS pathogenesis remains unclear. Previously, stomatin-like protein 2 (SLP2) and prohibitin (PHB) aggregates were identified in a model of *CHCHD10*-related ALS (*Chchd10^S59L/+^* mice). This study raises the question of the presence and possible involvement of these aggregates in ALS beyond *CHCHD10*-associated motor neuron disease (MND). Using immunohistofluorescence, we analyzed SLP2/PHB expression in the spinal MNs and hippocampus of two ALS mouse models: *Fus^ΔNLS^* and *Sod1^G86R^*. Additionally, post-mortem spinal cord tissues from 27 ALS and ALS-FTD patients were analyzed. SLP2/PHB aggregates were identified in spinal MNs and the hippocampus of *Fus^ΔNLS^* mice but not in *Sod1^G86R^* mice. In ALS patients, SLP2/PHB aggregation was observed in four cases, including two with *C9ORF72* mutations. Interestingly, aggregates were absent in *SOD1*-associated ALS patients. These findings suggest that SLP2/PHB aggregation is not specific to *CHCHD10* variants but may contribute to the pathogenesis of ALS from different origins. The age-related accumulation of these aggregates highlights their potential role in disease progression and as therapeutic targets. Future studies should investigate their mechanistic contributions across different ALS subtypes.

## 1. Introduction

Amyotrophic Lateral Sclerosis (ALS) is a fatal motor neuron disease (MND) characterized by the progressive degeneration of upper and lower motor neurons (MNs), ultimately leading to paralysis and death within 3 to 5 years after symptom onset [1]. ALS occurs in both sporadic (90–95%) and familial (5–10%) forms. The most frequent causative genes, responsible for more than 50% of familial ALS and about 7.5% of sporadic ALS, include *C9ORF72* (Hexanucleotide repeat expansion in the chromosome 9 open reading frame 72), *SOD1* (superoxide dismutase 1), *TARDBP* (encoding TDP-43, TAR DNA-binding protein 43), and *FUS* (Fused in Sarcoma) [2,3].

Clinically, ALS frequently overlaps with frontotemporal dementia (FTD), the second most common form of dementia after Alzheimer’s disease. ALS and FTD are now considered part of a continuous clinical spectrum: up to 50% of ALS patients display cognitive or behavioral symptoms indicative of frontal lobe involvement, while approximately 15% of FTD patients develop motor neuron signs. Shared variants in genes such as *C9ORF72*, *TARDBP*, and *FUS* further support the existence of overlapping pathogenic mechanisms [4,5]. FTD is a heterogeneous neurodegenerative disorder primarily affecting the frontal and anterior temporal lobes, leading to progressive impairments in cognition and behavior. To date, there is no effective disease-modifying treatments for ALS, FTD, or ALS-FTD, highlighting the need for a deeper understanding of the molecular pathways involved to identify new therapeutic strategies [6].

ALS and FTD also share neuropathological hallmarks, notably the accumulation of misfolded proteins forming cytoplasmic inclusions in degenerating neurons. These proteinopathies, including aggregates of TDP-43, *SOD1*, and *FUS*, highlight crucial dysregulation in protein homeostasis and cellular stress responses [7,8]. More recently, novel mitochondria-localized protein aggregates have emerged as potential contributors to disease mechanisms. Notably, aggregates of *CHCHD10*, a mitochondrial protein, have been described in the brain of FTD patients [9] as well as in cellular and mouse models expressing *CHCHD10* mutations [9,10,11,12,13,14,15].

Among the genetic forms of ALS-FTD, *CHCHD10* has been identified as a causative gene (ALS-FTD2, OMIM #615911), particularly in patients harboring the heterozygous *p.Ser59Leu* (*S59L*) variant [16,17,18,19,20]. *CHCHD10* encodes a mitochondrial intermembrane protein enriched at cristae junctions, where it contributes to mitochondrial structure and function. The identification of *CHCHD10* variants in ALS-FTD patients provided the first genetic evidence directly linking primary mitochondrial disfunction to MN degeneration. To investigate the pathogenic mechanisms associated with *CHCHD10* variants, we previously developed a *Chchd10^S59L/+^* knock-in mouse model, which recapitulates key clinical and pathological features of ALS-FTD [10,11]. These mice exhibit neuromuscular junction dysfunction, spinal MN degeneration, and TDP-43 cytoplasmic aggregates, consistent with the TDP-43 proteinopathy observed in ALS patients [11,12]. They also display hippocampal protein inclusions and cognitive impairments resembling FTD [10]. Notably, *CHCHD10* aggregates are observed in both spinal and hippocampal regions, some of which colocalize with phosphorylated TDP-43 aggregates [9,10].

Previously, we demonstrated that *CHCHD10* interacts with stomatin-like protein 2 (SLP2) [15], a mitochondrial scaffolding protein that stabilizes the prohibitin (PHB) complex, composed of PHB1 and PHB2, in the inner mitochondrial membrane [21]. In *Chchd10^S59L/+^* mice, we observed the formation of perinuclear SLP2/PHB aggregates in spinal MNs and smaller aggregates in the hippocampus [10,15]. These aggregates correlate with neuronal death, suggesting that abnormal clustering of mitochondrial scaffold proteins may contribute to neurodegeneration in *CHCHD10*-associated ALS-FTD [15]. However, whether mitochondrial SLP2/PHB aggregation represents a common pathological mechanism across ALS subtypes, beyond *CHCHD10-*related disease, remains unknown. This represents a critical knowledge gap in understanding the broader role of mitochondrial dysfunction in ALS pathogenesis.

Here, we investigated whether mitochondrial scaffold protein aggregation involving SLP2 and PHBs constitutes a shared pathological hallmark across genetically diverse ALS models and patient tissues, potentially uncovering novel biomarkers or therapeutic targets relevant to precision medicine approaches. To address this, we analyzed the presence of SLP2/PHB aggregates in two well-established ALS mouse models with distinct pathogenic mechanisms: the *Sod1^G86R^* transgenic model, which mimics toxic gain-of-function effects of mutant *SOD1* and is characterized by mitochondrial stress and oxidative damage [22,23]; and the *Fus^ΔNLS^* knock-in model, which presents a defective nuclear localization signal, leading to cytoplasmic *FUS* accumulation and downstream TDP-43 pathology [24,25]. These complementary models encompass both mitochondrial and RNA-binding protein-related mechanisms of MND, providing an ideal framework to determine whether SLP2/PHB aggregation is a common feature of ALS, independent of its genetic origin.

Finally, to assess the translational relevance of our findings, we extend our analysis to postmortem tissues from 27 ALS and ALS-FTD patients. Our results reveal that SLP2/PHB aggregates are also present in human ALS cases, regardless of genetic background. These findings suggest that the aggregation of mitochondrial scaffold protein represents a more general pathogenic mechanism in ALS than previously thought, with potential implications for diagnostic and therapeutic strategies.

## 2. Results

### 2.1. SLP2/PHB Aggregation in Fus^ΔNLS^ Mice but Not in the Sod1^G86R^ Model

**Figure 1:**
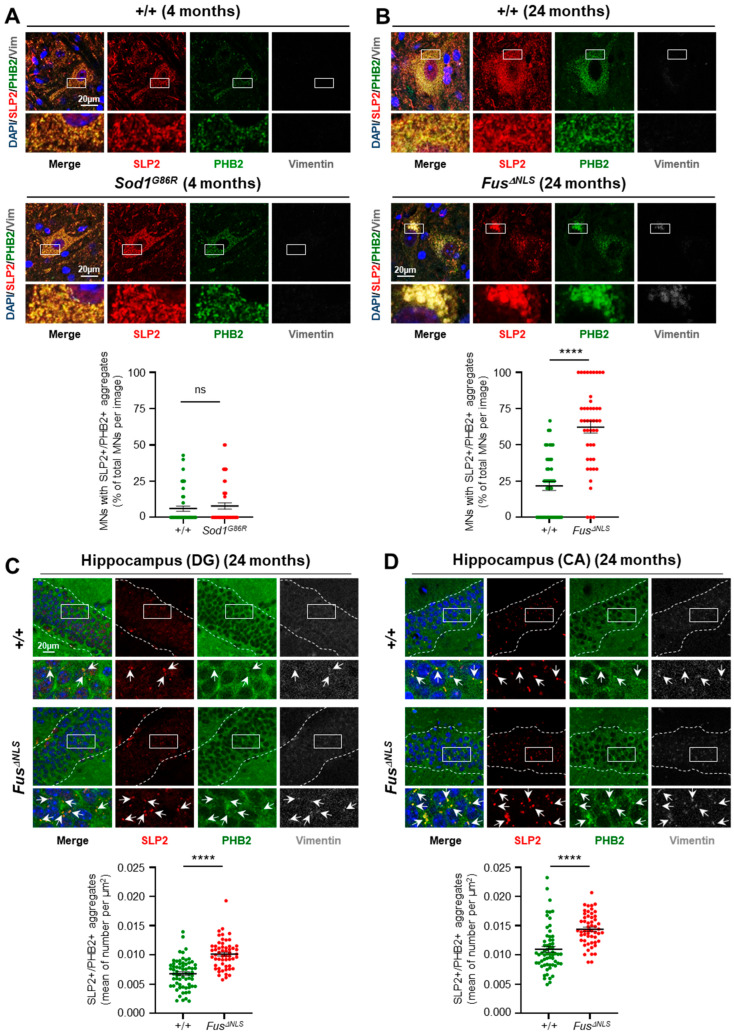
SLP2/PHB aggregation in the *Fus^ΔNLS^* mouse model, but not in *Sod1^G86R^* mice. (**A**) Representative lumbar spinal cord sections from end-stage *Sod1^G86R^* mice (4 months) and age-matched control (+/+) immunostained for SLP2 (red), PHB2 (green), and Vimentin (gray), DAPI (blue). No SLP2/PHB2 aggregated were detected. Scale bar = 20 µm. Quantification of MNs containing SLP2/PHB aggregates is shown below. (**B**) Lumbar spinal cord neurons from end-stage *Fus^ΔNLS^* mice (24 months) and age-matched control (+/+) showing large perinuclear aggregates immunolabelled with SLP2, PHB2, and Vimentin, consistent with aggresome-liked structures. Scale bar = 20 µm. Quantification of MNs with SLP2/PHB aggregates is shown below. (**A**,**B**) *n* = 3 mice per group, 15 images per animal. (**C**,**D**) Hippocampal sections of *Fus^ΔNLS^* mice showing prominent SLP2/PHB2 aggregation. Arrows indicate colocalized SLP2/PHB aggregates. Scale bars= 20 µm. Quantification of SLP2/PHB aggregates in the dentate gyrus (DG, **C**) and *cornus ammonis* (CA1-CA3, **D**) is shown below. Hippocampal analyses were performed on 3 *Fus^ΔNLS^* mice and 3 controls, with 3-5 randomly selected fields per region and 5 images per field. (**A**,**D**) Data are presented as mean ± SEM and were analyzed using the Mann–Whitney test. *p*-values: **** < 0.0001; ns= not significant.

Our research on *Chchd10^S59L/+^* mice demonstrated that SLP2 and PHB aggregates accumulate in degenerating spinal MNs and hippocampal neurons, correlating with neuronal loss [10,15]. This led us to investigate whether these aggregates are exclusive to *CHCHD10*-associated disease or found in ALS of other origins. To this end, we analyzed SLP2/PHB aggregates in two ALS mouse models: *Sod1^G86R^*, which exhibits a severe form as early as 4 months of age [22,23], and *Fus^ΔNLS^*, which develops a late-onset disease with symptoms at 24 months of age [24,25]. Contrary to our results in *Chchd10^S59L/+^* mice at the end-stage (around one year of age) [15], immunostaining for SLP2 and PHB2 did not reveal any abnormal expression in the spinal MNs of *Sod1^G86R^* mice at end-stage (4 months) (Figure 1A). However, large perinuclear aggregates expressing both SLP2 and PHB2 were observed in spinal MNs of *Fus^ΔNLS^* mice at 24 months of age (Figure 1B). These aggregates were also positive for vimentin, consistent with their aggresome-related nature. Quantitative analysis confirmed a significant increase in the proportion of MNs containing SLP2/PHB aggregates in *Fus^ΔNLS^* mice compared to controls. Furthermore, significant SLP2 and PHB2 aggregation was detected in *Fus^ΔNLS^* mice hippocampus (Figure 1C,D), mirroring the patterns observed in *Chchd10^S59L/+^* mice [15].

### 2.2. Aggregates Including SLP2 and PHB2 Are Also Present in ALS Patients

To extend the relevance of these findings to human disease, we analyzed a series of 27 patients diagnosed with ALS and ALS-FTD. This cohort included 14 individuals with sporadic ALS, aged between 54 and 74 years, 2 with sporadic ALS-FTD (aged 42 and 70), and 11 patients presenting with monogenic forms of ALS. Among the latter, there were 7 with *C9ORF72* mutations (5 diagnosed with ALS and 2 with ALS-FTD, aged 53 to 77), 3 with *SOD1* mutations (aged 50 to 73), and 1 patient with *TARBDP*-associated disease (aged 47) (Table 1). Additionally, we analyzed 3 control subjects (aged 84, 94, and 95), two of whom exhibited Braak stage III Alzheimer’s lesions.

**Figure 2:**
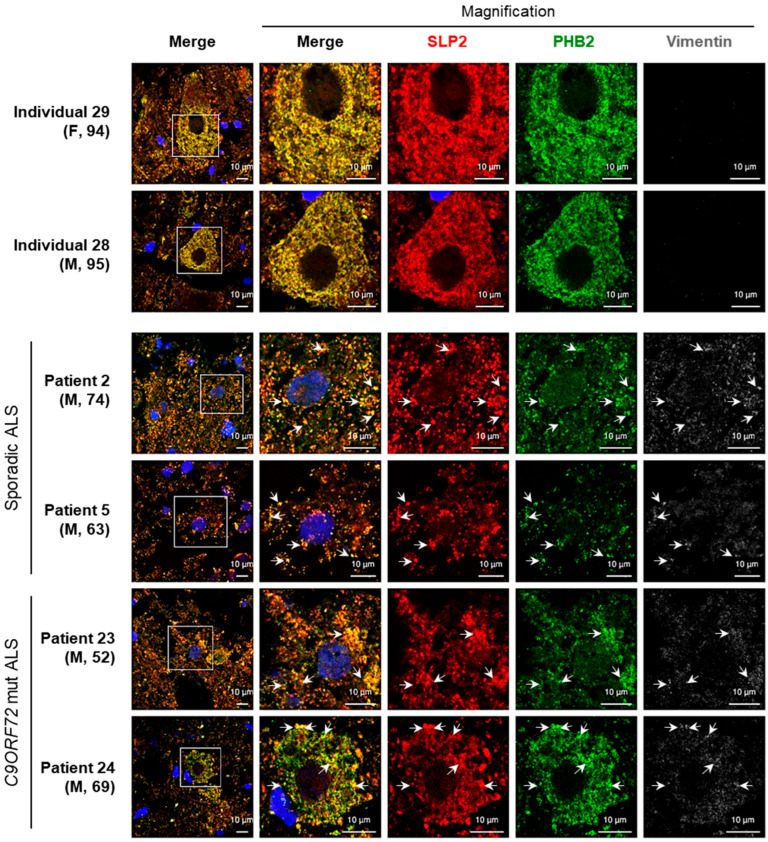
SLP2/PHB aggregates in spinal motor neurons of ALS patients. Representative spinal cord sections from two control individuals (29 and 28, aged 94 and 95 years), two sporadic ALS patients (2 and 5, aged 74 and 63 years), and two *C9ORF72*-associated ALS patients (23 and 24, aged 52 and 69 years). Sections were immunostained with antibodies against SLP2 (red), PHB2 (green), and Vimentin (gray), DAPI (blue). Arrows indicate colocalized SLP2/PHB aggregates. Scale bar = 10 µm. F = female; M = male.

We identified SLP2/PHB2 aggregates in spinal MNs of 2 patients with sporadic ALS (patients 2 and 5, aged 74 and 63, respectively) and 2 patients presenting a *C9ORF72*-associated ALS (patients 23 and 24, aged 52 and 69, respectively) (Figure 2). Quantitative analysis revealed a significant increase in the proportion of MNs containing SLP2/PHB aggregates in ALS patients compared to controls (Table 2). In sporadic ALS patients, 43–45% of MNs contained aggregates vs. 14–18% in controls. Similarly, in *C9ORF72*-associated ALS patients, 39–48% of MNs harbored aggregates, confirming a significant enrichment relative to control individuals. Notably, as for the *Sod1^G86R^* mouse model, these aggregates were absent in patients with *SOD1* variants. Taken together, these results suggest that SLP2/PHB aggregation is not specific to *CHCHD10*-associated disease and that SLP2 and prohibitins may be involved in the cascade of events leading to neuronal death in ALS of other origins.

## 3. Discussion

**Figure 3:**
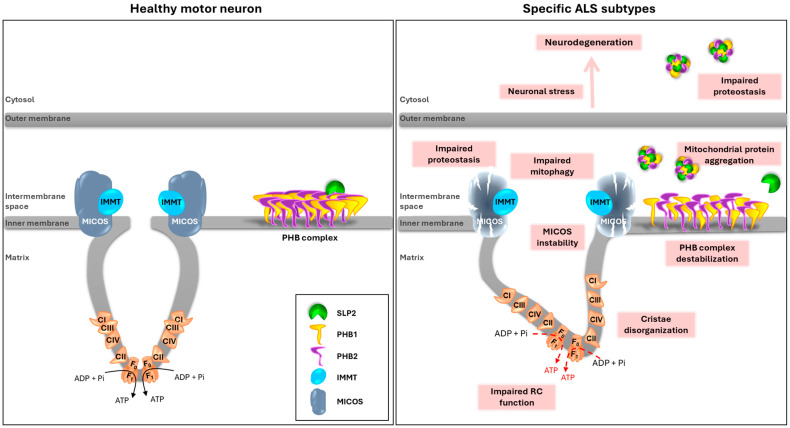
Proposed model of SLP2/PHB aggregation in ALS pathogenesis (adapted from [15]). Schematic representation illustrating how SLP2/PHB aggregation may contribute to ALS. In healthy MNs, SLP2 and PHB complex support, at least in part, mitochondrial homeostasis by maintaining cristae organization, respiratory chain (RC) integrity, and efficient mitophagy. In specific ALS subtypes (e.g., *CHCHD10*, *FUS* or *C9ORF72* mutations), destabilization of the PHB complex and/or MICOS disorganization may promote the formation of SLP2/PHB aggregates within mitochondria. These aggregates disrupt mitochondrial architecture and function, leading to defective mitophagy, impaired proteostasis, accumulation of cytoplasmic aggregates, and ultimately to neuronal stress and neurodegeneration.

This study provides the first evidence that SLP2/PHB protein aggregation may contribute to ALS pathogenesis beyond *CHCHD10* mutations, revealing a potentially broader role for mitochondrial scaffold protein dysregulation in MND (Figure 3).

### 3.1. SLP2/PHB Aggregation in the Fus^ΔNLS^ ALS Model

In the *Fus^ΔNLS^* knock-in mouse model, we identified large perinuclear SLP2/PHB aggresomes in spinal MNs and smaller aggregates in the hippocampus. Quantification revealed an approximately 190% increase in the spinal cord, 50% increase in the DG, and 30% increase in the CA region of *Fus^ΔNLS^* mice compared to controls. These findings closely mirror those previously observed in *Chchd10^S59L/+^* mouse model, where SLP2 and PHB form aggregates correlating with neurodegeneration, showing an approximate 210% increase in the spinal cord, 166% in DG, and 94% in CA at the end-stage of disease [10,15]. Importantly, the occurrence of such aggregates in *Fus^ΔNLS^* mice, harboring a mutation unrelated to mitochondrial genes, suggests that SLP2/PHB aggregation may be a downstream consequence of convergent neurodegenerative mechanisms, rather than a feature exclusive to *CHCHD10*-related disease.

### 3.2. Detection of SLP2/PHB Aggregates in ALS Patient Tissues

Consistent with this, we observed SLP2/PHB aggregates in spinal motor neurons of 4 ALS out of 27 ALS and ALS-FTD patients. Quantitative analysis showed that control individuals exhibited ~17.7% and ~14.3% of MNs containing SLP2/PHB aggregates, whereas two sporadic ALS patients displayed ~43.5% and ~45.1%, and two *C9ORF72* ALS patients showed ~47.6% and ~39.5% of MNs with SLP2/PHB aggregates. Although the morphology of these aggregates differed from those observed in mouse models, probably due to post-mortem tissue degradation, fixation artifacts, or disease stage, their presence in human tissues supports the clinical relevance and translational potential of our findings. These observations reinforce the hypothesis that mitochondrial scaffold protein aggregation may contribute to disease pathology in a subset of ALS patients, independent of the underlying genetic cause.

### 3.3. Absence of SLP2/PHB Aggregates in the Sod1^G86R^ ALS Model

Interestingly, no SLP2/PHB aggregates were observed in the *Sod1^G86R^* mouse model. This result is particularly relevant since we specifically selected the G86R variant, which (unlike the widely used G93A mutant) does not accumulate in mitochondria upon overexpression [26], thereby minimizing potential confounding effect related to abnormal mitochondrial localization of *SOD1*. The absence of SLP2/PHB aggregates in *Sod1^G86R^* mice, combined with their presence in only a minority of human ALS samples, suggests that SLP2/PHB aggregation is not a universal feature of ALS, but rather a pathological hallmark of specific molecular subtypes. This aligns with the known clinical and pathological heterogeneity of ALS; wherein different patients exhibit distinct proteinopathy profiles and cellular stress responses [27,28]. These findings highlight the importance of molecularly stratified approaches in both the study and treatment of ALS.

### 3.4. Age-Dependent Accumulation of SLP2/PHB Aggregates

Our data also suggest that SLP2/PHB aggregation may accumulate progressively with aging in mouse models. In *Chchd10^S59L/+^* mice, SLP2/PHB aggregates are absent at early stages (e.g., 3 months of age) but become increasingly prominent with age [15]. This progressive accumulation may reflect the gradual deterioration of mitochondrial function, impaired proteostasis, and reduced autophagic clearance, hallmarks of aging known to contribute to neurodegenerative processes [29,30,31]. In particular, the formation of SLP2/PHB aggregates may indicate a failure of mitophagy, the selective autophagic clearance of damaged mitochondria, which is increasingly recognized as a critical pathway disrupted in ALS and other neurodegenerative disorders [32,33]. Impaired autophagic flux or overload of the degradation system could contribute to the persistence and accumulation of these mitochondrial aggregates, further exacerbating neuronal stress and degeneration. Notably, we also detected these aggregates in a small number of MNs in aged control mice, further supporting the idea that aging may independently promote their formation. In human samples, however, the presence of SLP2/PHB aggregates was not restricted to the oldest individuals in the cohort (aged 52, 63, 69, and 74 years), suggesting that factors beyond chronological age, such as genetic predisposition or cumulative cellular stress, may influence aggregate formation.

### 3.5. Integrative Model of SLP2/PHB Aggregation in ALS Pathogenesis

Altogether, these results point to SLP2/PHB aggregation as a multifactorial phenomenon, influenced by both genetic mutations (e.g., *CHCHD10*, *FUS, C9ORF72*) and age-related cellular decline. The formation of these aggregates may represent a converging pathological mechanism affecting mitochondrial function, possibly through disruption of mitochondrial architecture, cristae dynamics, or inner membrane homeostasis. Moreover, our findings raise the possibility that SLP2/PHB aggregation reflects impaired mitophagic processes in certain ALS subtypes. Given the central role of autophagy in maintaining mitochondrial quality control, especially in post-mitotic neurons, future studies should explore whether modulating autophagic activity could prevent or reduce the accumulation of these aggregates. Targeting autophagy or mitophagy-related pathways may therefore represent a promising therapeutic avenue for ALS subtypes characterized by mitochondrial proteinopathy [34,35].

## 4. Materials and Methods

### 4.1. Mouse Models

*Sod1^G86R^* and *Fus^ΔNLS^* mice were generated as described in [24,25] and housed at Faculty of Medicine from Strasbourg University under controlled conditions (12/12 h light/dark cycle, 21 ± 1 °C, 60% humidity) with ad libitum access to food and water. *Fus^ΔNLS^* mice, expressing a truncated *FUS* protein lacking the PY-NLS (encoded by exon 15 of the *Fus* gene), were maintained in congenic C57Bl6/J. *Sod1^G86R^* mice (FVB-Tg(Sod1*G86R)M1Jwg/J, Jax Strain#005110) were maintained in a pure FVB/N background. To reduce animal use, tissues from previous studies were used [36,37]. All experiments were approved by the Strasbourg University ethical committee (CREMEAS, references 2016111716439395 and 25452). No inclusion or exclusion criteria were applied for animal selection, and no data points were excluded during analysis.

### 4.2. Immunohistofluorescence on Mouse Tissues

Immunostaining was performed on lumbar spinal cord sections from *Sod1^G86R^* and *Fus^ΔNLS^* mice and on brain sections from *Fus^ΔNLS^* mice as previously described [15]. Briefly, antigen retrieval was performed in citrate buffer (pH6.0; Vector Laboratories, H3300) for 15 min at 95 °C followed by 15 min at room temperature (RT). Brain or spinal cord sections were then incubated for 1 h at RT in blocking buffer containing 0.25–0.5% Triton X-100, 5% normal goat serum, and 4% bovine serum albumin (BSA). Floating sections were incubated overnight at 4 °C with primary antibodies diluted in blocking buffer: mouse anti-PHB2 (Proteintech, Rosemont, IL, USA, 66424-1-Ig; 1/400), rabbit anti-SLP2 (Proteintech, 10348-1-AP; 1/100), and goat anti-Vimentin (Merck, Darmstadt, Germany, V4360; 1/150). After several washes, sections were incubated for 1 h at RT in a solution of PBS containing 0.3% Triton with the corresponding Alexa Fluor-conjugated secondary antibodies: Alexa Fluor 488 (Invitrogen, (Thermo Fisher Scientific) Waltham, MA, USA, A11029; 1/500), Alexa Fluor 594 (Invitrogen, A21207; 1/500), and Alexa Fluor 647 (Invitrogen, A32849; 1/500). Nuclei were counterstained DAPI (2 µg/mL; Life Technologies (Thermo Fisher Scientific), Carlsbad, CA, USA) for 5 min at RT. Sections were then mounted using Vectashield Hard Set medium (Vector Laboratories, Newark, CA, USA).

Confocal images were acquired using a ZEISS LSM880 laser-scanning microscope. Aggregate quantification was performed using FIJI (ImageJ 1.54p; National Institutes of Health, Bethesda, MD, USA). Manual annotation of MNs containing SLP2/PHB aggregates was performed in a blinded manner with respect to genotype.

### 4.3. Immunohistofluorescence on Human ALS Tissues

Immunostaining on human lumbar spinal cord tissues was performed on 15 µm sections from 30 individuals, including 27 ALS patients and 3 controls (Table 1). Sections were fixed in 4% paraformaldehyde for 20 min, followed by antigen retrieval in 0.01M citrate buffer (pH 6) at 90 °C for 12 min. Samples were then permeabilized and blocked for 1 h in PBS containing 0.25% Triton X-100 (Sigma-Aldrich, Darmstadt, Germany), 4% bovine serum albumin, and 5% normal goat serum. Immunolabelling was performed using the same antibodies and dilutions as for mouse tissues.

To mitigate lipofuscin autofluorescence, sections were mounted using EverBrite™ TrueBlack^®^ Hardset mounting medium (Biotium, Fremont, CA, USA, #23018-T). Imaging was performed with a confocal laser scanning microscope (LSM880; Carl Zeiss, Oberkochen, Germany) to assess SLP2 and PHB expression. Manual annotation of MNs containing SLP2/PHB aggregates was performed in a blinded manner. Human spinal cord samples were provided from the Brainbank Neuro-CEB Neuropathology Network and the Pitié-Salpétrière Hospital, (Paris, France).

### 4.4. Statistical Analysis

Quantitative analysis of SLP2/PHB aggregates in lumbar spinal MNs of *Sod1^G86R^* and *Fus^ΔNLS^* mice, as well as in brain sections from *Fus^ΔNLS^* mice, was performed using GraphPad Prism 8.0 (GraphPad Software). Comparisons between two independent groups were conducted using the non-parametric Mann–Whitney U test. All analyses were two-tailed, and *p*-values < 0.05 were considered statistically significant.

For human spinal cord tissues, statistical analyses were performed on 4 ALS patients exhibiting SLP2/PHB aggregates in >30% of analyzed MNs and 2 control individuals (17–51 MNs per individual). The frequency distribution of MNs with (pathological) versus without (non-pathological) SLP2/PHB aggregates was compared between ALS patients and controls using the chi-square (χ^2^) test in GraphPad Prism 8.0 (GraphPad Software). *p*-values < 0.05 were considered statistically significant.

## 5. Conclusions

In conclusion, these results suggest that SLP2/PHB aggregation not only may reflect a structural disruption of mitochondrial homeostasis but may also be indicative of impaired mitochondrial turnover, potentially involving autophagy and mitophagy dysfunctions. Our findings expand the spectrum of proteinopathy in ALS to include mitochondrial scaffold protein aggregation, offering new insights into disease pathogenesis and highlighting potential therapeutic targets. Future work should aim to elucidate the molecular mechanisms by which SLP2 and PHB contribute to neuronal dysfunction and determine whether modulating their expression or preventing their aggregation could ameliorate mitochondrial dysfunction and neurodegeneration in ALS. Additionally, broader studies in genetically diverse patient cohorts will be essential to define the prevalence and clinical relevance of SLP2/PHB aggregation and to explore its utility as a biomarker for precision medicine approaches in ALS.

## Figures and Tables

**Table 1:** Clinical and genetic characteristics of the ALS and ALS-FTD cohort. The cohort comprised 27 patients, including 14 with sporadic ALS, 2 with sporadic ALS-FTD, and 11 with monogenic forms of ALS. Among the monogenic cases, 7 carried *C9ORF72* expansions, 3 had *SOD1* mutations, and 1 carried *TARDBP* mutation. Patients exhibiting SLP2/PHB2 aggregates are highlighted in bold. “?” indicates missing information for this patient.

Patient Number	ALS Form	Pathology	Sexe	Age of Death
1	ALS	sporadic	M	55
**2**	**ALS**	**sporadic**	**M**	**74**
3	ALS	sporadic	M	53
4	ALS	sporadic	M	69
**5**	**ALS**	**sporadic**	**M**	**63**
6	ALS	sporadic	F	62
7	ALS	sporadic	M	57
8	ALS	sporadic	F	66
9	ALS	sporadic	M	65
10	ALS	sporadic	?	?
11	ALS	sporadic	?	?
12	ALS-FTD	sporadic	M	42
13	ALS	sporadic	F	55
14	ALS	sporadic	?	?
15	ALS	sporadic	M	54
16	ALS-FTD	sporadic	M	70
17	ALS	*C9ORF72* mutation	M	63
18	ALS	*C9ORF72* mutation	F	60
19	ALS	familial *SOD1 D83G* mutation	M	73
20	ALS	familial *SOD1 D83G* mutation	M	58
21	ALS	*C9ORF72* mutation	M	53
22	ALS	*TARDBP G348V* mutation	M	47
**23**	**ALS**	***C9ORF72* mutation**	**M**	**52**
**24**	**ALS**	***C9ORF72* mutation**	**M**	**69**
25	ALS	*SOD1* mutation	M	50
26	ALS-FTD	*C9ORF72* mutation	M	66
27	ALS-FTD	*C9ORF72* mutation	F	77
28	Control	Braak stage III and Thal stage 2 Alzheimer lesions + amyloid angiopathy	M	95
29	Control	none	F	94
30	Control	Braak stage III Alzheimer lesions	M	84

**Table 2:** Comparison of MN without or with SLP2/PHB aggregates in ALS patients and control individuals. The table summarizes the percentage (%) and number (n) of lumbar spinal MNs exhibiting SLP2/PHB aggregates versus those without aggregates in 4 ALS patients with detectable aggregates and 2 control individuals. Only MNs analyzed in which >30% contained aggregates in ALS patients were included. Data provided a comparison between the frequency distribution of MNs with (pathological) and without (non-pathological) SLP2/PHB aggregates between ALS patients and controls using the chi-square (χ^2^) test.

Patient Number	ALS Form	Motor Neurons	% (n)	χ^2^
2	sporadic ALS	without aggregates	56.52 (13)	*p* < 0.0001
		with aggregates	43.48 (10)	
5	sporadic ALS	without aggregates	54.90 (28)	*p* < 0.0001
		with aggregates	45.10 (23)	
23	*C9ORF72* ALS	without aggregates	52.38 (11)	*p* < 0.0001
		with aggregates	47.62 (10)	
24	*C9ORF72* ALS	without aggregates	60.53 (23)	*p* < 0.0001
		with aggregates	39.47 (15)	
28	Control	without aggregates	85.71 (18)	*p* < 0.0001
		with aggregates	14.29 (3)	
29	Control	without aggregates	82.35 (14)	*p* < 0.0001
		with aggregates	17.65 (3)	

## Data Availability

The original contributions presented in this study are included in the article. Further inquiries can be directed to the corresponding author.

## Abbreviations

ALS	Amyotrophic Lateral Sclerosis
CA	*Cornus Ammonis*
*CHCHD10*	Coiled-coil-Helix-Coiled-coil-Helix Domain containing protein 10
*C9ORF72*	Chromosome 9 open reading frame 72
DG	Dentate Gyrus
FTD	Frontotemporal Dementia
*FUS*	Fused in Sarcoma
MN	motor neuron
MND	motor neuron disease
PHB	Prohibitin
SLP2	Stomatin-Like Protein 2
*SOD1*	Superoxide Dismutase
*TARDBP*	TAR DNA Binding Protein
